# High Rates of Biomarker-Confirmed Alcohol Use Among Pregnant Women Living With HIV in South Africa and Uganda

**DOI:** 10.1097/QAI.0000000000002156

**Published:** 2019-09-18

**Authors:** Greer A. Raggio, Christina Psaros, Robin Fatch, Georgia Goodman, Lynn T. Matthews, Jessica F. Magidson, Gideon Amanyire, Anna Cross, Stephen Asiimwe, Judith A. Hahn, Jessica E. Haberer

**Affiliations:** a Department of Psychiatry, Massachusetts General Hospital, Harvard Medical School, Boston, MA;; b National Center for Weight and Wellness, Washington, DC;; c Department of Medicine, University of California San Francisco, San Francisco, CA;; d Center for Global Health, Massachusetts General Hospital, Boston, MA;; e Department of Medicine, Massachusetts General Hospital, Harvard Medical School, Boston, MA;; f Department of Infectious Disease, University of Alabama at Birmingham, Birmingham, AL;; g Department of Psychology, University of Maryland, College Park, MD;; h Makerere University Joint AIDS Program, Kampala, Uganda;; i Desmond Tutu HIV Foundation, University of Cape Town, Cape Town, South Africa;; j Department of Community Health, Mbarara University of Science and Technology, Mbarara, Uganda; and; k Kabwohe Clinical Research Center, Kabwohe, Uganda.

**Keywords:** alcohol, HIV, pregnancy, South Africa, Uganda, phosphatidylethanol, women living with HIV

## Abstract

Alcohol use is common among people living with HIV and particularly harmful during pregnancy. However, objective data on alcohol use in pregnant women living with HIV (WLWH) are lacking. In areas with high levels of alcohol use generally, such as South Africa and Uganda, these data are needed to inform interventions.

**Methods:**

Pregnant and nonpregnant, antiretroviral therapy-naive WLWH were recruited from outpatient clinics in South Africa and Uganda. Women provided self-report data on previous three-month alcohol use and potential mental health correlates of alcohol use (depression and stigma). Blood samples were used to measure phosphatidylethanol (PEth), an objective biomarker of recent alcohol intake. We analyzed any alcohol use (ie, any self-reported use or PEth-positive [≥8 ng/mL]) and under-reporting of alcohol use (ie, no self-reported use with concurrent PEth-positive).

**Results:**

Among pregnant WLWH (n = 163, median age was 26 [interquartile range: 23–29], median gestational age was 20 weeks [interquartile range: 16–26]), 40% were using alcohol and 16% under-reported alcohol use. Neither any alcohol use nor under-reporting of alcohol use differed significantly between pregnant and nonpregnant women or by country (*P* > 0.05). Greater depression (but not greater stigma) was significantly associated with any alcohol use (adjusted odds ratio = 1.41, 95% confidence interval: [1.01 to 1.99]; *P* = 0.045).

**Conclusions:**

Alcohol use was prevalent and under-reported among pregnant WLWH in South Africa and Uganda, similar to nonpregnant participants, and associated with depression. General health care and antenatal clinic settings present opportunities to provide integrated alcohol-based counseling and depression treatment.

## Abstract 2

Supplemental Digital Content is Available in the Text.

## INTRODUCTION

Hazardous alcohol use is an ongoing problem among individuals living with HIV worldwide. In South Africa and Uganda, where HIV prevalence is among the highest in the world,^1^ the prevalence of hazardous alcohol use (ranging from heavy drinking to alcohol use disorders) is 7%–31% among persons living with HIV.^2–5^ Alcohol consumption among persons living with HIV is linked to lower adherence to antiretroviral therapy (ART) and, in some studies, compromised immunologic function and higher mortality.^6^

The harms of alcohol use are even greater among pregnant women living with HIV (WLWH), in part because of the adverse health impact on the developing fetus. Alcohol use during pregnancy is associated with low birth weight, preterm delivery, and fetal alcohol syndrome, the latter of which affects as many as 21% of children in the Western Cape of South Africa.^7–9^ Maternal HIV infection itself^10,11^ and exposure to some forms of ART^12^ may further increase risk for some of these poor outcomes.

Studies of perinatal alcohol use in South Africa and Uganda describe self-reported intake ranging from 18% to 43% during pregnancy.^13–16^ Although many WLWH may reduce or cease alcohol intake after learning of their pregnancy,^16,17^ women who drink during pregnancy often do so at hazardous levels. Desmond and colleagues^16^ found that, among pregnant WLWH in KwaZulu-Natal, South Africa, two-thirds of drinkers (12% of the full sample) reported typically consuming 3 or more drinks on each drinking occasion. Another analysis found that 9% of pregnant WLWH recruited in Cape Town, South Africa reported hazardous drinking while being aware of their pregnancy^18^; these mothers' infants were more likely to be small for gestational age than those born to women reporting low levels of alcohol intake during pregnancy.

Perinatal alcohol use in South Africa and Uganda has not been evaluated using objective biomarkers measured during pregnancy, representing a significant gap in the literature for several reasons. For one, nonstandard portion sizing^19^ and varied ethanol content of alcoholic beverages^20^ across sub-Saharan Africa makes self-report data in aggregate inherently unreliable. Moreover, persons living with HIV may under-report alcohol consumption because of social desirability bias, or fear of being denied ART by medical providers.^21^ Indeed, studies comparing self-reported to objectively measured alcohol use among nonpregnant persons living with HIV in Uganda reveal under-estimates of both frequency and amount of alcohol use,^22,23^ and a 50% under-reporting rate among patients initiating ART and testing positive for objective alcohol use.^24^ Pregnant WLWH may be especially motivated to conceal unhealthy or risky behaviors, such as alcohol consumption. A comparison of self-reported and objectively measured alcohol intake among pregnant WLWH, therefore, represents a critical next step in the literature. Moreover, gathering these objective data is particularly important in areas with known high per capita alcohol consumption, such as South Africa and Uganda.^25^

Understanding the drivers of alcohol use among pregnant WLWH is also important, and a necessary precursor to developing interventions aimed at reducing perinatal alcohol intake. Pregnancy is often a stressful time for women; one qualitative study among pregnant women surveyed at South African drinking establishments noted participants “drink[ing] [their] problems away,” suggesting the use of alcohol to deal with complex life challenges.^26^ This stress may be magnified among pregnant WLWH, who experience the added burdens of managing HIV, potential for HIV transmission to the newborn, and concerns about future child-rearing capacity.^17,27^ Depression is twice as common among pregnant WLWH compared with HIV-seronegative pregnant women in sub-Saharan Africa,^28,29^ and has been linked to increased self-reported alcohol use among pregnant WLWH in South Africa.^16^ Relatedly, overt discrimination by health care workers due to HIV stigma has been reported by pregnant WLWH in Uganda and identified as a driver of maladaptive health behaviors, such as suboptimal engagement in HIV care.^30^ It remains unknown whether HIV-related stigma is associated with alcohol use among pregnant WLWH in South Africa or Uganda.

The aims of this paper are to: (1) quantify alcohol consumption and under-reporting of alcohol consumption using self-report and objective biomarker data among pregnant WLWH starting ART in South Africa and Uganda; (2) compare any alcohol consumption and under-reporting of alcohol consumption between pregnant and nonpregnant WLWH; and (3) evaluate HIV-related depression and HIV-related stigma as correlates of alcohol consumption among pregnant and nonpregnant WLWH, with pregnancy status as a potential effect modifier.

## METHODS

### Recruitment and Participants

**FIGURE 1.**
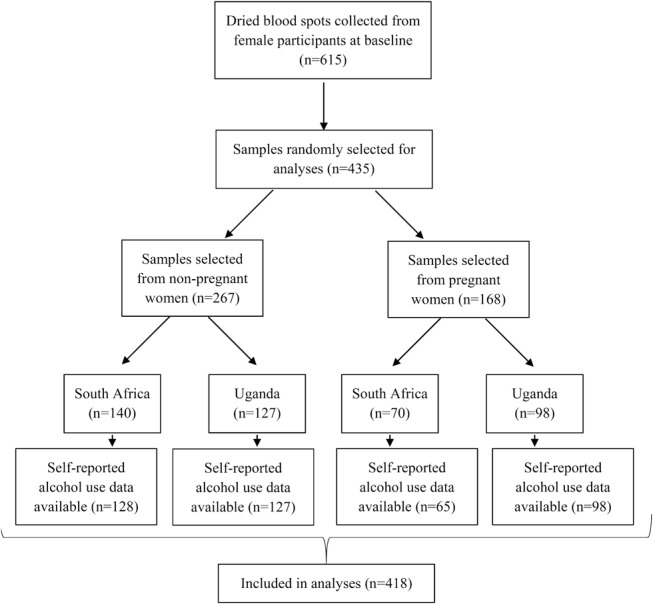
Testing flow for study of WLWH in South Africa and Uganda.

The results presented in this paper represent cross-sectional, secondary analyses from a longitudinal study of adherence to ART among individuals with HIV initiating treatment (Measuring Early Treatment Adherence [META]; ClinicalTrials.gov, NCT02419066^31^). Participants were recruited from outpatient clinics offering HIV care in Cape Town, South Africa, and southwestern Uganda between March 2015 and September 2016. Patients were approached by study staff upon presenting for care. To be eligible for the parent study, participants were required to meet the following criteria: (1) diagnosed with HIV; (2) ART-naive; (3) initiating ART within one month of enrollment; (4) at least 18 years old; (5) live within 60 km of the clinic; (6) intending to stay in the area for the next year; (7) if pregnant, no more than 34 weeks gestation; (8) able to provide informed consent; and (9) able to communicate in English or local languages (Runyankole or Xhosa). Participants were excluded if they had either: (1) intermediate stage disease at enrollment (CD4: 200–349); or (2) among pregnant women only, late stage disease (CD4 < 200). For the analyses reported here, the sample was restricted to baseline visits only, and to individuals who provided both PEth and self-report data (Fig. 1). All women received care according to national guidelines for ART treatment. The threshold for ART initiation was a CD4 count <500 cells/mm^3^ during the recruitment period in both sites.

### Measures

Participants completed a structured interview (measures detailed below) and blood draw at enrollment. Urine samples were collected from all women below age 50 to confirm pregnancy status.

#### Demographics

Participants were asked to provide basic demographic information including but not limited to age, income, marital status, and educational achievement.

#### HIV and Pregnancy Characteristics

Participants were asked to provide time since HIV diagnosis and gestational age. Past 3-month CD4 cell count was extracted from participant medical charts, if available, and otherwise assessed by study blood draw at enrollment.

#### Alcohol Consumption

Self-reported and objective alcohol consumption were measured using the 3-item Alcohol Use Disorders Identification Test—Consumption (AUDIT-C)^32^ and a blood biomarker (phosphatidylethanol [PEth]), respectively. For the present study, we modified the AUDIT-C to account for only the past 3 months of alcohol intake (versus past year). The AUDIT-C is scored on a scale of 0–12, with higher scores representing greater self-reported alcohol intake.

To measure objective alcohol use, PEth results were obtained by testing dried blood spots prepared from venous blood draws. PEth levels reflect alcohol intake up to 3 weeks before blood draw.^33^ All samples were tested at the U.S. Drug Testing Laboratories in Des Plaines, IL using previously published methods.^34^ PEth testing has demonstrated good sensitivity and specificity (88.0% and 88.5%, respectively) in detecting any alcohol consumption among people living with HIV.^35^ PEth results have also effectively differentiated between true abstention and light, moderate, and heavy drinking among pregnant women.^36^

For the primary analyses in this paper, we defined alcohol use based on a combination of AUDIT-C and PEth values; a participant was positive for any alcohol use with an AUDIT-C > 0 (ie, some self-reported alcohol use) and/or PEth ≥ 8 ng/mL (ie, the lower limit of quantification). Under-report of alcohol use was defined as indication of no alcohol intake via self-report (AUDIT-C = 0) paired with a quantifiable PEth result (≥8 ng/mL). Heavy/hazardous alcohol use was defined as an AUDIT-C ≥ 3 (ie, self-report cutoff for hazardous drinking, among women specifically) and/or PEth ≥ 50 ng/mL, consistent with cutoffs for heavy drinking used in previous studies.^37^

#### Depression

Depressive symptoms were measured using the Depression Scale of the Hopkins Symptom Checklist (DHSCL).^38^ The scale used in the present study consisted of 12 items, each representing a distinct depressive symptom. This version of the DHSCL specifically omitted somatic symptoms of depression, as they may overestimate depression among individuals living with HIV.^39–41^ Participants were asked to rate each symptom on a 4-point scale based on the extent to which they experienced it over the previous week (1 = Not at all, 4 = Extremely). The DHSCL has successfully been used to assess depression prevalence among residents of rural Uganda^42^ and pregnant women across Africa.^43^ We used a cutoff of ≥ 1.75, which has demonstrated good sensitivity for detecting depression in studies among African populations.^44,45^

In addition to the DHSCL, pregnant participants completed the Edinburgh Postnatal Depression Scale.^46^ The Edinburgh Postnatal Depression Scale is a frequently used measure of perinatal depression severity and probable postnatal depression in African countries, including Uganda.^47^ Meta-analytic analyses have revealed a pooled sensitivity of 0.94 (95% confidence interval [CI]: 0.68 to –0.99) and a pooled specificity of 0.77 (95% CI: 0.59 to 0.88) at the cutoff score of ≥9.^43^ We used a cutoff score of ≥10, consistent with previous studies.^48,49^

#### Stigma

Perceived HIV-related stigma was measured using 20 items adapted from the Berger HIV Stigma Scale.^50^ Items are scored on a 4-point Likert scale (1 = Strongly disagree, 4 = Strongly agree) and summed across 2 domains: perceived negative attitudes, and disclosure concerns. Higher scores indicate greater perceived HIV-related stigma.

### Statistical Analyses

For descriptive analyses, we calculated proportions for categorical variables, and means, SDs, medians, and interquartile ranges (IQRs) for continuous variables. We examined bivariate associations between participant characteristics by pregnancy status and by country (separately) using χ^2^ tests (categorical variables) and Mann–Whitney tests (continuous variables). We conducted logistic regression to test bivariate associations between each independent variable of interest (pregnancy status; depression; perceived negative attitudes stigma; and disclosure concerns stigma), sociodemographics, and any alcohol use (yes/no; primary outcome variable). Multivariable logistic regression models were used to test the associations of these variables with any alcohol use, while controlling for the following demographic covariates selected a priori: age, marital status, education, and study site. We additionally examined pregnancy as a potential effect modifier of these associations using interaction terms in separate multivariable models, with *P* < 0.10 on the likelihood ratio test considered statistically significant.

## RESULTS

### Participant Characteristics

Descriptive statistics for demographic and clinical characteristics are presented in Table 1. Female study participants randomly selected for PEth testing (n = 435) were similar to those who were not selected for PEth testing (n = 180; 32 pregnant, 148 not pregnant); no significant differences were observed in age, marital status, education, household assets, months since HIV diagnosis, depression, or the disclosure concerns stigma scale. CD4 count was significantly higher among participants who had PEth results compared with those without (median = 428 [IQR: 193–516], versus 384 [IQR: 144–451]; *P* < 0.01). Finally, scores on the perceived negative attitudes stigma scale were significantly lower among those with PEth results compared with those without (median = 1 [IQR: 0–2], versus 2 ([IQR: 0–3]; *P* = 0.02).

**TABLE 1.**
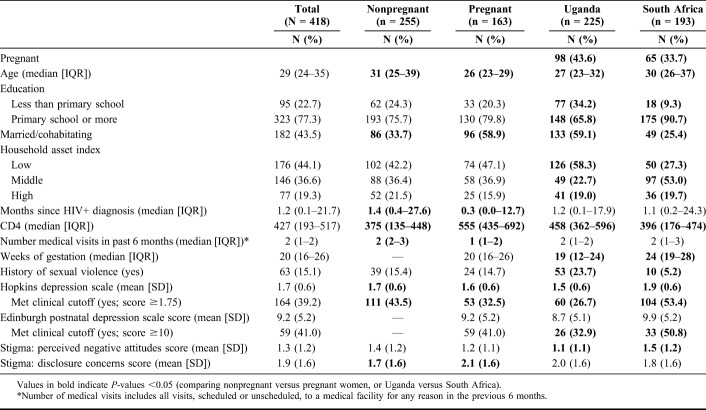
Participant Demographic and Clinical Characteristics: Overall, by Group, and by Study Site (N = 418)

### Alcohol Use Prevalence

Table 2 presents descriptive statistics for alcohol use, and comparisons of alcohol use across participant groups based on pregnancy status and country. Among the 418 participants, 28.5% self-reported any alcohol use in the past 3 months (AUDIT-C >0). In comparison, 37.8% met criteria for any objective alcohol intake based on biomarker results (PEth ≥8 ng/mL). Combining self-report and objective alcohol use data, 42.6% (n = 178) of all participants met criteria for any alcohol use (AUDIT-C >0 and/or PEth ≥8 ng/mL), with 14.1% of women under-reporting alcohol use. Furthermore, 27.0% (n = 113) met criteria for heavy/hazardous alcohol use based on self-report and PEth results.

**TABLE 2.**
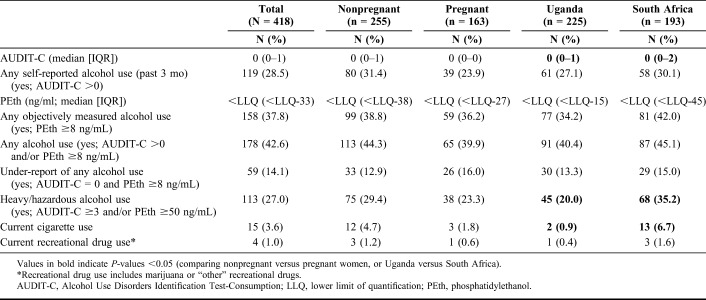
Participant Substance Use: Overall, by Group, and by Study Site (N = 418)

Comparing participants based on pregnancy status, 39.9% of pregnant participants were positive for any alcohol use (versus 44.3% nonpregnant), and 23.3% were positive for heavy/hazardous alcohol use (versus 29.4% nonpregnant). A greater proportion of pregnant women under-reported current alcohol use (16.0% pregnant versus 12.9% nonpregnant). None of these differences were statistically significant (*P* > 0.05). Compared by study site, significantly more participants in South Africa met criteria for heavy/hazardous drinking (35.2%) compared with those in Uganda (20.0%; *P* < 0.01).

### Correlates of Alcohol Use

Tables 3 and 4 present results of multivariable analyses examining pregnancy and depression, respectively, as correlates of any alcohol use. Pregnancy status was not significantly associated with alcohol use in bivariate or multivariable analyses, (adjusted Odds Ratio [aOR] = 0.80, 95% CI: [0.51 to 1.24], *P* = 0.32 [Table 3]); the association remained nonsignificant in bivariate analyses stratified for age, marital status, and study site (data not shown). Higher depression scores (on the DHSCL) were, however, significantly associated with any alcohol use after adjusting for pregnancy status and demographic covariates (age, marital status, education, and study site; aOR = 1.41, 95% CI: [1.01 to 1.99], *P* = 0.045 [Table 4]). Pregnancy status was not a statistically significant effect modifier of this relationship in the full sample (interaction *P* > 0.10); however, when we stratified these analyses by study site, pregnancy was a significant effect modifier among Ugandan participants only (depression + pregnant, aOR = 2.25, 95% CI: [1.02 to 4.94], *P* = 0.04 [see Table, Supplemental Digital Content, http://links.lww.com/QAI/B376]). Neither stigma type (disclosure concerns stigma or negative attitudes stigma) was significantly associated with alcohol use in multivariable analyses (disclosure concerns, OR = 0.98, 95% CI: [0.87 to 1.11], *P* = 0.78; negative attitudes, OR = 0.97, 95% CI: [0.82 to 1.15], *P* = 0.72), and pregnancy status was not a statistically significant effect modifier in these relationships (disclosure concerns + not pregnant, aOR = 1.04, 95% CI: [0.89 to 1.21]; disclosure concerns + pregnant, aOR = 0.90, 95% CI: [0.74 to 1.10]; negative attitudes + not pregnant, aOR = 1.07, 95% CI: [0.87 to 1.33]; negative attitudes + pregnant, aOR = 0.81, 95% CI: [0.60 to 1.08]; all interactions, *P* > 0.10).

**TABLE 3.**
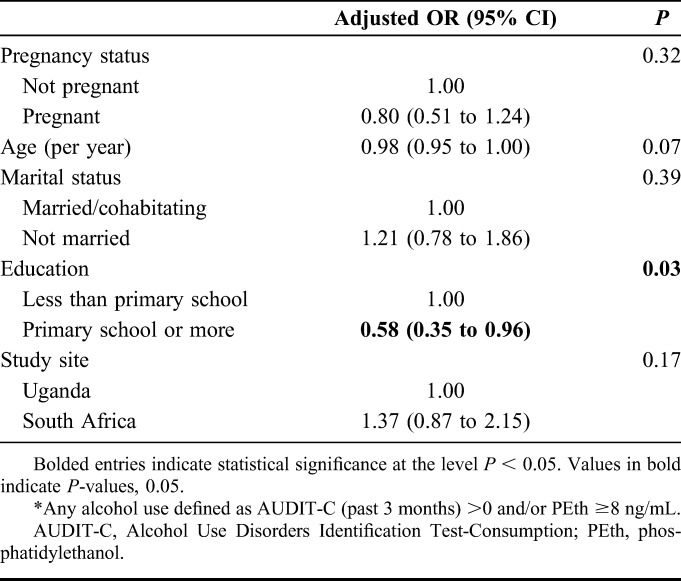
Multivariable Logistic Regression Model Examining the Association Between Pregnancy Status and Any Alcohol Use (N = 418)*

**TABLE 4.**
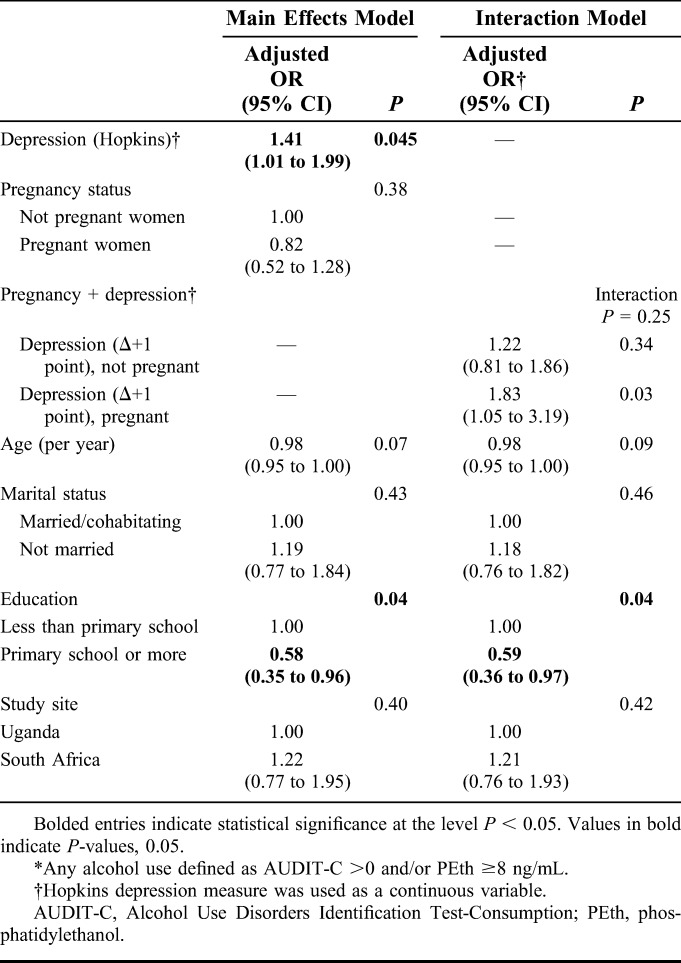
Multivariate Analyses Testing Depression as a Correlate of Any Alcohol Use and Pregnancy as a Moderator of This Relationship (N = 418)*

## DISCUSSION

We used a sensitive alcohol biomarker, phosphatidylethanol (PEth), to demonstrate a 43% prevalence of alcohol consumption among WLWH initiating ART in South Africa and Uganda. The inclusion of both pregnant and nonpregnant WLWH is unique in this study and allowed for direct comparisons of alcohol use and under-reporting of alcohol use across groups.

The high rates of perinatal alcohol use (40% any use, 23% heavy/hazardous use) among pregnant WLWH in our study are alarming, and exceed most previously reported figures,^16,18^ including biological measures of ethanol use drawn from newborn meconium.^51^ Our use of objective and sensitive biomarker data to quantify alcohol intake likely accounts for some of the observed discrepancy, and provided a more accurate assessment of alcohol use compared with self-report. Furthermore, close to one in 4 pregnant WLWH engaged in heavy/hazardous perinatal drinking, suggesting high risk for adverse infant outcomes in our sample. Heavy episodic drinking during pregnancy is associated with poor pregnancy outcomes (eg, low birth weight) and risk for long-term neurodevelopmental deficits in children (eg, attention problems), some of which persist into adulthood.^7–9,52^ Knowledge of the risk of poor pregnancy outcomes associated with alcohol consumption should be reinforced among all pregnant women, and support provided to avoid any amount of alcohol intake. Indeed, data on the impact of light-to-moderate perinatal drinking on fetal neurodevelopment are inconclusive, and no level of alcohol use during pregnancy is considered reliably safe.^53,54^ In addition, given emerging data on poorer birth outcomes among WLWH and exposed to ART,^12,55^ our findings highlight the importance of objectively measuring other exposures known to affect birth outcomes.

Notably, alcohol use among pregnant WLWH in our study was not statistically different from nonpregnant women (40% versus 44%, respectively). This finding raises questions about women's motivation and/or ability to cease alcohol intake after pregnancy recognition, and the role of cultural factors such as social drinking norms. Although we did not collect data on alcohol use preceding conception, previous research has identified prepregnancy alcohol consumption as a strong predictor of perinatal consumption among women in South Africa^56^; this suggests that alcohol use counseling and reduction interventions could best support women of child-bearing age if offered before conception in addition to during pregnancy. For example, alcohol-based counseling could be integrated in women's health care, HIV care, and antenatal care settings, and may be performed by health care providers during clinic visits or off-site through community-based support groups.^57–59^

A significant minority of participants under-reported their alcohol intake, with similar rates demonstrated by pregnant and nonpregnant women (16% versus 13%, respectively). Our findings are consistent with a Ugandan study showing a 13% rate of alcohol use under-reporting among nonpregnant WLWH.^24^ Some under-report of alcohol use is unsurprising given social desirability bias,^60^ and that individuals initiating ART may be motivated to underplay their drinking behaviors for fear of being denied treatment.^21^ In fact, the rate of alcohol use under-reporting among pregnant WLWH in our study is surprisingly low. This finding may suggest a pre-established atmosphere of open communication among patients and providers in surveyed clinics or, conversely, patients' willingness to disclose alcohol use to study research assistants specifically (but not necessarily to medical providers), as observed in previous research.^23^ The former explanation may be supported by the low levels of HIV-related stigma reported by our participants. As a third and final explanation, many WLWH in our study may have been unaware of the dangers posed by alcohol use during pregnancy, giving them little reason to conceal their true alcohol intake. Future research in this area should evaluate the association, if any, between women's knowledge of the impact of alcohol consumption on birth outcomes and objective alcohol intake.

A significantly greater proportion of our study participants (pregnant and nonpregnant) recruited from the South African clinic were heavy/hazardous drinkers (35%) compared with women in Uganda (20%). This discrepancy is consistent with population-based data showing more prevalent heavy episodic drinking in South Africa (10.4%) compared with Uganda (3.4%).^61^ We did not explore the underpinnings of this finding; however, multifaceted cultural differences between countries, including social drinking norms, likely play a part.^26,62^ Future research should identify key factors associated with alcohol consumption in each country and, based on those data, develop alcohol reduction interventions tailored to local WLWH.

Depression was common and found in approximately 40% of all WLWH in the study. Depression was also significantly associated with alcohol use, such that WLWH with greater depressive symptoms were more likely to consume alcohol. These depression rates are consistent with the extant literature^29,63^ and suggestive of a pervasive problem among WLWH with potentially serious consequences. Indeed, a systematic review of the literature among persons living with HIV in sub-Saharan Africa showed that depressed patients were 55% less likely to demonstrate good adherence to ART compared to their nondepressed peers.^64^ Interventions that integrate depression treatment with alcohol-based counseling are needed. Furthermore, although pregnancy status was not a statistically significant effect modifier of the relationship between depression and alcohol use in our complete participant sample, we did find a significant association between incremental increases in depression and alcohol use among pregnant women only; it is possible that our relatively small sample size may have limited statistical power to detect a significant result in the full participant pool. Further research should attempt to confirm these findings in a larger sample.

Some methodological limitations to our study should be noted. First, PEth biomarker data reflect only sustained alcohol intake up to 3 weeks before blood draws,^65^ whereas the self-report measure captures alcohol use from the preceding 3 months. This mismatch may have affected the accuracy of our findings by underestimating the rate of under-reporting. Thus, our estimate of under-reporting in this sample of WLWH is likely a conservative one. Second, our findings may have differed had we prospectively measured alcohol intake in the months following ART initiation (ie, after study enrollment). Research suggests that individuals starting HIV treatment are often driven to reduce alcohol intake in the short-term; however, alcohol reduction efforts may wane over time, possibly in the absence of HIV- or ART-related physical symptoms.^37^ Indeed, an analysis of data from men and WLWH recruited for our parent study showed heavier alcohol use among individuals with higher CD4 levels in Uganda.^66^ Third, it is possible that PEth results among pregnant WLWH in our study were skewed because of intake of traditional fermented food and beverages with low, but detectable, alcohol content, and that are believed to render health benefits during pregnancy.^14^ Fourth, our participant sample had significantly higher (ie, better) CD4 levels and lower reported stigma compared with individuals from the parent study not included in these analyses. It is possible, therefore, that our data would not generalize to all persons living with HIV in the geographic regions sampled. Fifth, it is worth noting that some pregnant participants meeting criteria for perinatal alcohol consumption may have done so only before learning of their pregnancy. In other words, women who drank during pregnancy may not have been aware of their pregnancy. Those who have stopped drinking since learning they are pregnant may need counseling to avoid pregnancy during times of heavy alcohol use in the future, but may not need counseling during the current pregnancy. To maximize the efficacy of alcohol use interventions for pregnant WLWH, women should be asked about the timing of their drinking behaviors in relation to pregnancy recognition. Finally, the generalizability of our findings may be limited by the exclusion of individuals with intermediate stage disease, and the short average duration of time since HIV diagnosis (1.2 months) in our sample. The latter may have altered our results in a number of ways, namely inflating depression scores, and potentially decreasing alcohol use.^37^

### Conclusions and Future Directions

This study is the first to provide biomarker-corroborated data on alcohol consumption among pregnant WLWH in sub-Saharan Africa, and to examine differences by pregnancy status across 2 African countries. We show very high proportions of WLWH using alcohol, with no difference by pregnancy. Depression was also common among both pregnant and nonpregnant participants, and positively associated with alcohol intake.

Offering integrated alcohol-based counseling and mental health services in women's HIV care and antenatal care settings may improve HIV health status and promote healthier pregnancies. The potential utility of integrating these services may be understood through a syndemics framework, wherein depression and alcohol use among WLWH represent intersecting epidemics with shared social and behavioral drivers.^9,67–69^ Services could be performed by health care providers and held during clinic visits or off-site. Community-based support and task shifting (ie, recruiting unspecialized or untrained health workers to conserve resources) show promise for large-scale delivery of supportive services. The Mentor Mother Program, which trains community health workers to deliver home-visiting support and education to pregnant women in African countries, exemplifies this model.^70^

To minimize alcohol use under-reporting, providers should be encouraged to use a patient-centered, nonjudgmental approach in conversations about alcohol use with WLWH to encourage open dialogue and increase the likelihood of positive change. Future studies should also use biomarker data to corroborate alcohol use during pregnancy, and collect data on adverse pregnancy outcomes (eg, neurodevelopmental problems) to assess the health impact of objective alcohol intake among pregnant WLWH, particularly in settings with high alcohol use.

Finally, although some data support the use of PEth to distinguish levels of drinking (eg, none versus heavy) among pregnant women,^36,71^ more studies are needed to confirm the validity of this method in the context of pregnancy (and the physiological changes associated with pregnancy, such as increased blood volume, which could affect test results).

## Supplementary Material

SUPPLEMENTARY MATERIAL

## References

[1] UNAIDS. South Africa Factsheet: HIV and AIDS Estimates. 2016 Available at: http://www.unaids.org/en/regionscountries/countries/southafrica/. Accessed March 1, 2019.

[2] Kekwaletswe C Morojele N Nkosi S Depression, Alcohol Use and Adherence to Antiretroviral Therapy (ART). Poster presented at the: 6th International AIDS Society Conference on HIV Pathogenesis and Treatment; July 17–20, 2011; Rome, Italy.

[3] Myer L Smit J Le Roux L Common mental disorders among HIV-infected individuals in South Africa: prevalence, predictors, and validation of brief psychiatric rating scales. AIDS Patient Care STDS. 2008;22:147–158. 18260806 10.1089/apc.2007.0102

[4] Nakimuli-Mpungu E Munyaneza G Depression, alcohol abuse, and disclosure of HIV serostatus among rural HIV-positive individuals in western Uganda. Neurobehav HIV Med. 2011;2011:19–25.

[5] Olley BO Seedat S Stein DJ Persistence of psychiatric disorders in a cohort of HIV/AIDS patients in South Africa: a 6-month follow-up study. J Psychosom Res. 2006;61:479–484. 17011355 10.1016/j.jpsychores.2006.03.010

[6] Williams EC Hahn JA Saitz R Alcohol use and human immunodeficiency virus (HIV) infection: current knowledge, implications, and future directions. Alcohol Clin Exp Res. 2016;40:2056–2072. 27696523 10.1111/acer.13204 PMC5119641

[7] Donald KAM Fernandez A Claborn K The developmental effects of HIV and alcohol: a comparison of gestational outcomes among babies from South African communities with high prevalence of HIV and alcohol use. AIDS Res Ther. 2017;14:28. 28482927 10.1186/s12981-017-0153-z PMC5422965

[8] May PA Blankenship J Marais AS Approaching the prevalence of the full spectrum of fetal alcohol spectrum disorders in a South African population-based study. Alcohol Clin Exp Res. 2013;37:818–830. 23241076 10.1111/acer.12033 PMC3610844

[9] Russell B Eaton L Petersen-Williams P Intersecting epidemics among pregnant women: alcohol use, interpersonal violence, and HIV infection in South Africa. Curr HIVAIDS Rep. 2013;10:103–110. 10.1007/s11904-012-0145-5 PMC3572769 23233038

[10] Slyker JA Patterson J Ambler G Correlates and outcomes of preterm birth, low birth weight, and small for gestational age in HIV-exposed uninfected infants. BMC Pregnancy Childbirth. 2014;14:7. 24397463 10.1186/1471-2393-14-7 PMC3897882

[11] Turner AN Tabbah S Mwapasa V Severity of maternal HIV-1 disease is associated with adverse birth outcomes in Malawian women: a cohort study. J Acquir Immune Defic Syndr. 2013;64:392–399. 23846560 10.1097/QAI.0b013e3182a2d13c PMC3940209

[12] Zash R Jacobson DL Diseko M Comparative safety of antiretroviral treatment regimens in pregnancy. JAMA Pediatr. 2017;171:e172222. 28783807 10.1001/jamapediatrics.2017.2222 PMC5726309

[13] Croxford J Viljoen D Alcohol consumption by pregnant women in the Western Cape. S Afr Med J. 1999;89:962–965. 10554632

[14] Namagembe I Jackson LW Zullo MD Consumption of alcoholic beverages among pregnant urban Ugandan women. Matern Child Health J. 2010;14:492–500. 19629663 10.1007/s10995-009-0500-3 PMC2906221

[15] Vythilingum B Roos A Faure SC Risk factors for substance use in pregnant women in South Africa. S Afr Med J. 2012;102:851–854. 23116742 10.7196/samj.5019

[16] Desmond K Milburn N Richter L Alcohol consumption among HIV-positive pregnant women in KwaZulu-Natal, South Africa: prevalence and correlates. Drug Alcohol Depend. 2012;120:113–118. 21820252 10.1016/j.drugalcdep.2011.07.004 PMC3223298

[17] Brittain K Remien RH Phillips T Factors associated with alcohol use prior to and during pregnancy among HIV-infected pregnant women in Cape Town, South Africa. Drug Alcohol Depend. 2017;173:69–77. 28199918 10.1016/j.drugalcdep.2016.12.017 PMC5429399

[18] Sania A Brittain K Phillips TK Effect of alcohol consumption and psychosocial stressors on preterm and small-for-gestational-age births in HIV-infected women in South Africa: a cohort study. BMJ Open. 2017;7:e014293. 10.1136/bmjopen-2016-014293 PMC5372146 28320796

[19] Hahn JA Bwana MB Javors MA Biomarker testing to estimate under-reported heavy alcohol consumption by persons with HIV initiating ART in Uganda. AIDS Behav. 2010;14:1265–1268. 20697796 10.1007/s10461-010-9768-y PMC2974914

[20] Francis JM Grosskurth H Kapiga SH Ethanol concentration of traditional alcoholic beverages in northern Tanzania. J Stud Alcohol Drugs. 2017;78:476–477. 28499118 10.15288/jsad.2017.78.476

[21] Papas R Gakinya B Baliddawa J Ethical issues in a stage 1 cognitive-behavioral therapy feasibility study and trial to reduce alcohol use among HIV-infected outpatients in western Kenya. J Empir Res Hum Res Ethics. 2012;7:29–37. 22850141 10.1525/jer.2012.7.3.29 PMC3690819

[22] Asiimwe SB Fatch R Emenyonu NI Comparison of traditional and novel self-report measures to an alcohol biomarker for quantifying alcohol consumption among HIV-infected adults in sub-Saharan Africa. Alcohol Clin Exp Res. 2015;39:1518–1527. 26148140 10.1111/acer.12781 PMC4515166

[23] Muyindike W Lloyd-Travaglini C Fatch R Phosphatidylethanol confirmed alcohol use among ART-naïve HIV-infected persons who denied consumption in rural Uganda. AIDS Care. 2017;29:1442–1447. 28278568 10.1080/09540121.2017.1290209 PMC5554736

[24] Bajunirwe F Haberer JE Boum Y II Comparison of self-reported alcohol consumption to phosphatidylethanol measurement among HIV-infected patients initiating antiretroviral treatment in southwestern Uganda. PLoS One. 2014;9:e113152. 25436894 10.1371/journal.pone.0113152 PMC4249861

[25] World Health Organization. Global Status Report on Alcohol and Health. 2018 Available at: http://apps.who.int/iris/bitstream/handle/10665/274603/9789241565639-eng.pdf?ua=1. Accessed March 1, 2019.

[26] Watt MH Eaton LA Choi KW “It's better for me to drink, at least the stress is going away”: perspectives on alcohol use during pregnancy among South African women attending drinking establishments. Soc Sci Med. 2014;116:119–125. 24997441 10.1016/j.socscimed.2014.06.048 PMC4117814

[27] Sandelowski M Barroso J Motherhood in the context of maternal HIV infection. Res Nurs Health. 2003;26:470–482. 14689463 10.1002/nur.10109

[28] Bernatsky S Souza R De Jong K Mental health in HIV-positive pregnant women: results from Angola. AIDS Care. 2007;19:674–676. 17505929 10.1080/09540120601012705

[29] Kaida A Matthews LT Ashaba S Depression during pregnancy and the postpartum among HIV-infected women on antiretroviral therapy in Uganda. J Acquir Immune Defic Syndr. 2014;67(suppl 4):S179–S187. 25436816 10.1097/QAI.0000000000000370 PMC4251908

[30] Ashaba S Kaida A Coleman JN Psychosocial challenges facing women living with HIV during the perinatal period in rural Uganda. PLoS One. 2017;12:e0176256. 28459866 10.1371/journal.pone.0176256 PMC5411062

[31] Haberer JE Bwana BM Orrell C ART adherence and viral suppression are high among most non-pregnant individuals with early-stage, asymptomatic HIV infection: An observational study from Uganda and South Africa. J Int AIDS Soc. 2019;22:e25232. 30746898 10.1002/jia2.25232 PMC6371013

[32] Bradley K Bush K Epler A Two brief alcohol-screening tests from the alcohol use disorders identification test (AUDIT): validation in a female veterans affairs patient population. Arch Intern Med. 2003;163:821–829. 12695273 10.1001/archinte.163.7.821

[33] Hahn JA Anton RF Javors MA The formation, elimination, interpretation, and future research needs of phosphatidylethanol for research studies and clinical practice. Alcohol Clin Exp Res. 2016;40:2292–2295. 27716960 10.1111/acer.13213 PMC5117827

[34] Jones J Jones M Plate C The detection of 1-palmitoyl-2-oleoyl-sn-glycero-3-phosphoethanol in human dried blood spots. Anal Methods. 2011;3:1101–1106.

[35] Hahn JA Dobkin LM Mayanja B Phosphatidylethanol (PEth) as a biomarker of alcohol consumption in HIV positives in sub-Saharan Africa. Alcohol Clin Exp Res. 2012;36:854–862. 22150449 10.1111/j.1530-0277.2011.01669.x PMC3310261

[36] Kwak H Han J Choi J Characterization of phosphatidylethanol blood concentrations for screening alcohol consumption in early pregnancy. Clin Toxicol Phila. 2014;52:24–31. 10.3109/15563650.2013.859263 24400931

[37] Hahn J Emenyonu N Fatch R Declining and rebounding unhealthy alcohol consumption during the first year of HIV care in rural Uganda, using phosphatidylethanol to augment self-report. Addiction. 2016;111:272–279. 26381193 10.1111/add.13173 PMC4715487

[38] Derogatis LR Lipman RS Rickels K The Hopkins Symptom Checklist (HSCL): a self-report symptom inventory. Behav Sci. 1974;19:1–15. 4808738 10.1002/bs.3830190102

[39] Martinez P Andia I Emenyonu N Alcohol use, depressive symptoms and the receipt of antiretroviral therapy in southwest Uganda. AIDS Behav. 2008;12:605–612. 17968651 10.1007/s10461-007-9312-x PMC3591721

[40] Kalichman SC Sikkema KJ Somlai A Assessing persons with human immunodeficiency virus (HIV) infection using the Beck Depression Inventory: disease processes and other potential confounds. J Assess. 1995;64:86–100. 10.1207/s15327752jpa6401_5 7877094

[41] Kalichman SC Rompa D Cage M Distinguishing between overlapping somatic symptoms of depression and HIV disease in people living with HIV-AIDS. J Nerv Ment Dis. 2000;188:662–670. 11048815 10.1097/00005053-200010000-00004

[42] Bolton P Wilk CM Ndogoni L Assessment of depression prevalence in rural Uganda using symptom and function criteria. Soc Psychiatry Psychiatr Epidemiol. 2004;39:442–447. 15205728 10.1007/s00127-004-0763-3

[43] Tsai AC Scott JA Hung KJ Reliability and validity of instruments for assessing perinatal depression in African settings: systematic review and meta-analysis. PLoS One. 2013;8:e82521. 24340036 10.1371/journal.pone.0082521 PMC3858316

[44] Chorwe-Sungani G Chipps J Validity and utility of instruments for screening of depression in women attending antenatal clinics in Blantyre district in Malawi. Afr Fam Pr. 2018;60:114–120. 10.4102/sajpsychiatry.v24i0.1181 PMC6295954 30568841

[45] Kaaya S Smith Fawzi M Mbwambo J Validity of the Hopkins symptom checklist-25 amongst HIV-positive pregnant women in Tanzania. Acta Psychiatr Scand. 2002;106:9–19. 12100343 10.1034/j.1600-0447.2002.01205.x PMC6300056

[46] Cox JL Holden JM Sagovsky R Detection of postnatal depression. Development of the 10-item Edinburgh postnatal depression scale. Br J Psychiatry. 1987;150:782–786. 3651732 10.1192/bjp.150.6.782

[47] Kakyo T Muliira J Mbalinda S Factors associated with depressive symptoms among postpartum mothers in a rural district in Uganda. Midwifery. 2011;28:374–379. 21601966 10.1016/j.midw.2011.05.001

[48] Leung SS Leung C Lam TH Outcome of a postnatal depression screening programme using the Edinburgh Postnatal Depression Scale: a randomized controlled trial. J Public Health (Oxf). 2011;33:292–301. 20884642 10.1093/pubmed/fdq075

[49] Ongeri L Wanga V Otieno P Demographic, psychosocial and clinical factors associated with postpartum depression in Kenyan women. BMC Psychiatry. 2018;18:318. 30285745 10.1186/s12888-018-1904-7 PMC6167779

[50] Berger BE Ferrans CE Lashley FR Measuring stigma in people with HIV: psychometric assessment of the HIV stigma scale. Res Nurs Health. 2001;24:518–529. 11746080 10.1002/nur.10011

[51] English L Mugyenyi G Nightingale I Prevalence of ethanol use among pregnant women in southwestern Uganda. Matern Child Health J. 2016;20:2209–2215. 27299903 10.1007/s10995-016-2025-x

[52] Behnke M Smith VC Prenatal substance abuse: short- and long-term effects on the exposed fetus. Pediatrics. 2013;131:e1009–e1024. 23439891 10.1542/peds.2012-3931 PMC8194464

[53] Jones KL The effects of alcohol on fetal development. Birth Defects Res C Embryo Today. 2011;93:3–11. 21425437 10.1002/bdrc.20200

[54] Polańska K Jurewicz J Hanke W Smoking and alcohol drinking during pregnancy as the risk factors for poor child neurodevelopment—a review of epidemiological studies. Int J Occup Med Env Health. 2015;28:419–443. 26190723 10.13075/ijomeh.1896.00424

[55] Uthman OA Nachega JB Anderson J Timing of initiation of antiretroviral therapy and adverse pregnancy outcomes: a systematic review and meta-analysis. Lancet HIV. 2017;4:e21–e30. 27864000 10.1016/S2352-3018(16)30195-3

[56] Choi KW Abler LA Watt MH Drinking before and after pregnancy recognition among South African women: the moderating role of traumatic experiences. BMC Pregnancy Childbirth. 2014;14:97. 24593175 10.1186/1471-2393-14-97 PMC3975846

[57] Milligan K Niccols A Sword W Maternal substance use and integrated treatment programs for women with substance abuse issues and their children: a meta-analysis. Subst Abuse Treat Prev Pol. 2010;5:21. 10.1186/1747-597X-5-21 PMC2942813 20809957

[58] Niccols A Milligan K Sword W Integrated programs for mothers with substance abuse issues: a systematic review of studies reporting on parenting outcomes. Harm Reduct J. 2012;9:14. 22429792 10.1186/1477-7517-9-14 PMC3325166

[59] Sweeney PJ Schwartz RM Mattis NG The effect of integrating substance abuse treatment with prenatal care on birth outcome. J Perinatol. 2000;20:219–224. 10879333 10.1038/sj.jp.7200357

[60] Del Boca FK Darkes J The validity of self-reports of alcohol consumption: state of the science and challenges for research. Addiction. 2003;98(suppl 2):1–12. 10.1046/j.1359-6357.2003.00586.x 14984237

[61] World Health Organization. Global Status Report on Alcohol and Health. 2014:128, 132 Available at: http://apps.who.int/iris/bitstream/handle/10665/112736/9789240692763_eng.pdf;jsessionid=7910DA48EE00E0EB292B71E37DEC9E9F?sequence=1. Accessed December 18, 2018. Accessed March 1, 2019.

[62] Kakufo A Bukuluki P Qualitative Research in Uganda on Knowledge, Attitudes and Practices Concerning Alcohol. Health Communication Partnership (HCP), Afford Project, Young Empowered and Healthy (YEAH). 2008 Available at: https://www.k4health.org/sites/default/files/Alcohol%20Study%20Report%20FINAL%20March%2013th.pdf.

[63] Yeji F Klipstein-Grobusch K Newell M Are social support and HIV coping strategies associated with lower depression in adults on antiretroviral treatment? Evidence from rural KwaZulu-Natal, South Africa. AIDS Care. 2014;26:1482–1489. 24991994 10.1080/09540121.2014.931561

[64] Nakimuli-Mpungu E Bass JK Alexandre P Depression, alcohol use and adherence to antiretroviral therapy in Sub-Saharan Africa: a systematic review. AIDS Behav. 2012;16:2101–2118. 22116638 10.1007/s10461-011-0087-8

[65] Wurst FM Thon N Yegles M Ethanol metabolites: their role in the assessment of alcohol intake. Alcohol Clin Exp Res. 2015;39:2060–2072. 26344403 10.1111/acer.12851

[66] Magidson J Fatch R Orrell C Biomarker-measured unhealthy alcohol use in relation to CD4 count among individuals starting ART in sub-Saharan Africa. AIDS Behav. 2019;23:1656–1667. 30560484 10.1007/s10461-018-2364-2 PMC6535416

[67] Pitpitan EV Kalichman SC Eaton LA Co-occurring psychosocial problems and HIV risk among women attending drinking venues in a South African township: a syndemic approach. Ann Behav Med. 2013;45:153–162. 23054944 10.1007/s12160-012-9420-3 PMC3578969

[68] Wong M Myer L Zerbe A Depression, alcohol use, and stigma in younger versus older HIV-infected pregnant women initiating antiretroviral therapy in Cape Town, South Africa. Arch Womens Ment Health. 2017;20:149–159. 27815628 10.1007/s00737-016-0688-3 PMC5500299

[69] Singer M Clair S Syndemics and public health: reconceptualizing disease in bio-social context. Med Anthr Q. 2003;17:423–441. 10.1525/maq.2003.17.4.423 14716917

[70] Rotheram-Borus MJ le Roux IM Tomlinson M Philani Plus (+): a mentor mother community health worker home visiting program to improve maternal and infants' outcomes. Prev Sci. 2011;12:372–388. 21850488 10.1007/s11121-011-0238-1 PMC3907085

[71] Bracero LA Maxwell S Nyanin A Improving screening for alcohol consumption during pregnancy with phosphatidylethanol. Reprod Toxicol. 2017;74:104–107. 28939493 10.1016/j.reprotox.2017.09.007

