# Supplementary material for: High Rates of Biomarker-Confirmed Alcohol Use Among Pregnant Women Living With HIV in South Africa and Uganda
Source: J Acquir Immune Defic Syndr. 2019 Sep 18;82(5):443–51. doi: 10.1097/QAI.0000000000002156 (PMC6857734; doi:10.1097/QAI.0000000000002156)
Supplement: SUPPLEMENTARY MATERIAL [file qai-82-443-s001.docx]

| **Supplementary Table. Multivariate analyses testing depression as a correlate of any alcohol use and pregnancy as a moderator of this relationship, stratified by study site (*N* = 418)*** | | | | | | | | | |
| --- | --- | --- | --- | --- | --- | --- | --- | --- | --- |
|  | Uganda (*n* = 225) | | | | South Africa (*n* = 193) | | | | |
|  | Adjusted OR  (95% CI) | *p*-value | Adjusted OR^×^  (95% CI) | *p*-value | Adjusted OR  (95% CI) | *p*-value | Adjusted OR^×^  (95% CI) | *p*-value |  |
| Depression (Hopkins) ^×^ | 1.19 (0.74, 1.89) | 0.47 | - |  | 1.79 (1.06, 3.01) | **0.03** | - |  |  |
| Pregnancy status |  | 0.83 |  |  |  | 0.18 |  |  |  |
| Not pregnant women | 1.00 |  | - |  | 1.00 |  | - |  |  |
| Pregnant women | 0.94 (0.50, 1.75) |  | - |  | 0.62 (0.31, 1.24) |  | - |  |  |
| Pregnancy + depression^×^ |  |  |  | intxn **p=0.04** |  |  |  | intxn p=0.91 |  |
| Depression (Δ+1 point), not pregnant | - |  | 0.80 (0.44, 1.47) | 0.48 | - |  | 1.83 (0.96, 3.49) | 0.07 |  |
| Depression (Δ+1 point), pregnant | - |  | 2.25 (1.02, 4.94) | 0.04 | - |  | 1.72 (0.72, 4.10) | 0.22 |  |
| Age (per year) | 1.00 (0.96, 1.03) | 0.90 | 1.00 (0.97, 1.04) | 0.93 | 0.95 (0.92, 0.99) | **0.01** | 0.95 (0.92, 0.99) | **0.01** |  |
| Marital status |  | 0.50 |  | 0.43 |  | 0.51 |  | 0.51 |  |
| Married/cohabitating | 1.00 |  | 1.00 |  | 1.00 |  | 1.00 |  |  |
| Not married | 1.23 (0.67, 2.27) |  | 1.28 (0.69, 2.37) |  | 1.26 (0.63, 2.51) |  | 1.26 (0.63, 2.51) |  |  |
| Education |  | 0.20 |  | 0.26 |  | 0.05 |  | 0.05 |  |
| Less than primary school | 1.00 |  | 1.00 |  | 1.00 |  | 1.00 |  |  |
| Primary school or more | 0.69 (0.39, 1.22) |  | 0.71 (0.40, 1.28) |  | 0.35 (0.12, 1.01) |  | 0.35 (0.12, 1.01) |  |  |

*Any alcohol use defined as AUDIT-C >0 and/or PEth ≥8 ng/ml

^×^ Hopkins depression measure was used as continuous variable

Definitions: AUDIT-C = Alcohol Use Disorders Identification Test – Consumption; PEth = phosphatidylethanol
